# Evaluation of Elevated Mean Pulmonary Arterial Pressure Based on Magnetic Resonance 4D Velocity Mapping: Comparison of Visualization Techniques

**DOI:** 10.1371/journal.pone.0082212

**Published:** 2013-12-12

**Authors:** Ursula Reiter, Gert Reiter, Gabor Kovacs, Aurelien F. Stalder, Mehmet A. Gulsun, Andreas Greiser, Horst Olschewski, Michael Fuchsjäger

**Affiliations:** 1 Division of General Radiology, Department of Radiology, Medical University of Graz, Graz, Austria; 2 Siemens AG, Healthcare Sector, Graz, Austria; 3 Division of Pulmology, Department of Internal Medicine, Medical University of Graz & LBI for Lung Vascular Research, Graz, Austria; 4 Siemens AG, Healthcare Sector, Erlangen, Germany; 5 Siemens Corporate Research, Baltimore, United States; University Hospital of Würzburg, Germany

## Abstract

**Purpose:**

Three-dimensional (3D) magnetic resonance phase contrast imaging (PC-MRI) allows non-invasive diagnosis of pulmonary hypertension (PH) and estimation of elevated mean pulmonary arterial pressure (mPAP) based on vortical motion of blood in the main pulmonary artery. The purpose of the present study was to compare the presence and duration of PH-associated vortices derived from different flow visualization techniques with special respect to their performance for non-invasive assessment of elevated mPAP and diagnosis of PH.

**Methods:**

Fifty patients with suspected PH (23 patients with and 27 without PH) were investigated by right heart catheterization and time-resolved PC-MRI of the main pulmonary artery. PC-MRI data were visualized with dedicated prototype software, providing 3D vector, multi-planar reformatted (MPR) 2D vector, streamline, and particle trace representation of flow patterns. Persistence of PH-associated vortical blood flow (t_vortex_) was evaluated with all visualization techniques. Dependencies of t_vortex_ on visualization techniques were analyzed by means of correlation and receiver operating characteristic (ROC) curve analysis.

**Results:**

t_vortex_ values from 3D vector visualization correlated strongly with those from other visualization techniques (r = 0.98, 0.98 and 0.97 for MPR, streamline and particle trace visualization, respectively). Areas under ROC curves for diagnosis of PH based on t_vortex_ did not differ significantly and were 0.998 for 3D vector, MPR vector and particle trace visualization and 0.999 for streamline visualization. Correlations between elevated mPAP and t_vortex_ in patients with PH were r = 0.96, 0.93, 0.95 and 0.92 for 3D vector, MPR vector, streamline and particle trace visualization, respectively. Corresponding standard deviations from the linear regression lines ranged between 3 and 4 mmHg.

**Conclusion:**

3D vector, MPR vector, streamline as well as particle trace visualization of time-resolved 3D PC-MRI data of the main pulmonary artery can be employed for accurate vortex-based diagnosis of PH and estimation of elevated mPAP.

## Introduction

Pulmonary hypertension (PH) is a life-threatening complex pathophysiological condition characterized by mean pulmonary arterial pressure (mPAP) equal to or exceeding 25 mmHg at rest [1], [2]. It is associated with vortical motion of blood in the main pulmonary artery [3], which can be evaluated from time-resolved 3-dimensional (3D) phase contrast magnetic resonance imaging (PC-MRI). The duration of PH-associated vortical blood motion correlates well with invasively obtained measurements of elevated mPAP. Thus, 3D PC-MRI of the main pulmonary artery is a reliable, non-invasive, non-ionizing screening and longitudinal tool for follow-up of patients with PH [3], [4].

Visualization of 3D velocity fields is crucial for detecting flow patterns in general and vortices in particular, but it remains challenging. Densely scattered in volume, 3D velocity vectors overlap and obscure each other, hampering analysis of regional flow patterns. To improve the visualization of 3D velocity fields, various approaches have been introduced that selectively reduce overall 3D information [5]–[7]. Post-processing techniques commonly applied to visualize 3D PC-MRI data represent velocities as 3D vectors or multi-planar reformatted (MPR) 2-dimensional (2D) vectors restricted to anatomic slices [8]–[11], or show calculated 3D time-varying integral curves such as streamlines or particle traces. Streamlines, defined as tangent curves to velocity vectors at a particular time point, describe instantaneous velocity directions in the 3D volume [8], [11], [12]; particle traces show the trajectories of particles moving in the 3D velocity field, providing a time-integrated picture of flow [11], [13].

The use of 3D vector representation of PC-MRI data to assess vortical blood motion in the main pulmonary artery and thereby diagnose PH and estimate elevated mPAP has been investigated previously [3]. Other flow visualization techniques, however, might be advantageous for vortex detection. It remains unknown whether PH-associated vortical blood motion can be evaluated from 2D MPR vector, streamline, or particle trace representation and how findings from 3D vector visualization might relate to findings from these commonly used PC-MRI visualization techniques.

The purpose of the present study was to compare the presence and duration of PH-associated vortical motion of blood in the main pulmonary artery on MPR vector, streamline, and particle trace visualization with results obtained from 3D vector visualization with special respect to their performance for non-invasive assessment of elevated mPAP and diagnosis of PH.

## Materials and Methods

### Study Population and Right Heart Catheterization

The prospective study was approved by the local ethical review board of the Medical University of Graz, Austria, and all subjects gave written informed consent. Subjects with known contraindications to MR were not enrolled. Fifty-three patients with known or suspected PH underwent PC-MRI of the main pulmonary artery after successful right heart catheterization (RHC). Three patients were excluded from evaluation due to inadequate PC-MRI data (one patient with severe arrhythmia, two patients with claustrophobia). Time delay between the two investigations of the remaining fifty patients was 10±14 days. No clinically relevant changes in drug treatment or disease state occurred between the two examinations.

RHC was performed with a 7F quadruple-lumen, balloon-tipped, flow directed Swan-Ganz catheter (Baxter Healthcare Corp, Irvine, CA, USA) using transjugular approach. Measurements were obtained in free breathing with the patient in the supine position. Invasively obtained mPAP was used as the reference standard for the diagnosis of PH [2]. The RHC-based classification and demographic characteristics of the study population are summarized in Table 1.

**Table 1:** Demographic characteristics of the study population.

parameter	total	patients ith PH	patients without PH
**No. of patients**	50	23	27
**No. of female/male patients**	34/16	13/10	21/6
**age (years)**	58±13 (23–82)	59±13 (23–82)	56±13 (27–77)
**mPAP at rest (mmHg)**	27±15 (8–58)	41±11 (26–58)	16±4 (8–24)

± standard deviations and ranges (minimum, maximum). Parameters are given as means

### MR Imaging

MR imaging was performed at 1.5 T (MAGNETOM Sonata, Siemens, Erlangen, Germany) using a 6-channel cardiac array coil, with the patient in the supine position. PC-MRI data were acquired in free breathing in right ventricular outflow tract (RVOT) orientation; the main pulmonary artery was covered in 5–10 gapless slices of a retrospectively ECG-gated, segmented, 2D spoiled gradient-echo-based phase contrast sequence with three-directional velocity encoding by a simple four-point velocity encoding scheme [14]. Velocity encoding (VENC) was set to 90 cm/s in all directions and adapted if necessary to prevent aliasing in the main pulmonary artery. Further protocol parameters were as follows: Field of view, 234–276×340 mm^2^; matrix, 96–114×192; slice thickness, 6 mm; bandwidth, 451 Hz/pixel; GRAPPA (generalized auto-calibrating partially parallel acquisition) factor, 2; number of reference lines, 21–26; flip angle, 15°; echo time, 4.1 ms; repetition time, 7.5 ms. 3 acquired phase encoding steps per segment resulted in a temporal resolution of 89 ms, which was interpolated to 20 cardiac phases per cardiac cycle. Three-fold averaging to suppress breathing artifacts yielded a measurement time of 66–72 heart beats per slice.

### Image Processing and Analysis

For calculation and visualization of 3D velocity fields, PC-MRI data were imported into dedicated prototype software (4D Flow, Siemens, Erlangen, Germany) [15], [16]. After background phase correction [13], [17], [18] and semi-automatic segmentation of the RVOT and main pulmonary artery, blood flow patterns were analyzed in 3D vector, MPR vector, streamline, and particle trace representations by two experienced readers who were blinded to mPAP measurements derived from RHC. First, the readers evaluated blood flow patterns independently so that interobserver variability could be assessed. Subsequently, they evaluated blood flow patterns in consensus.

**Figure 1:**
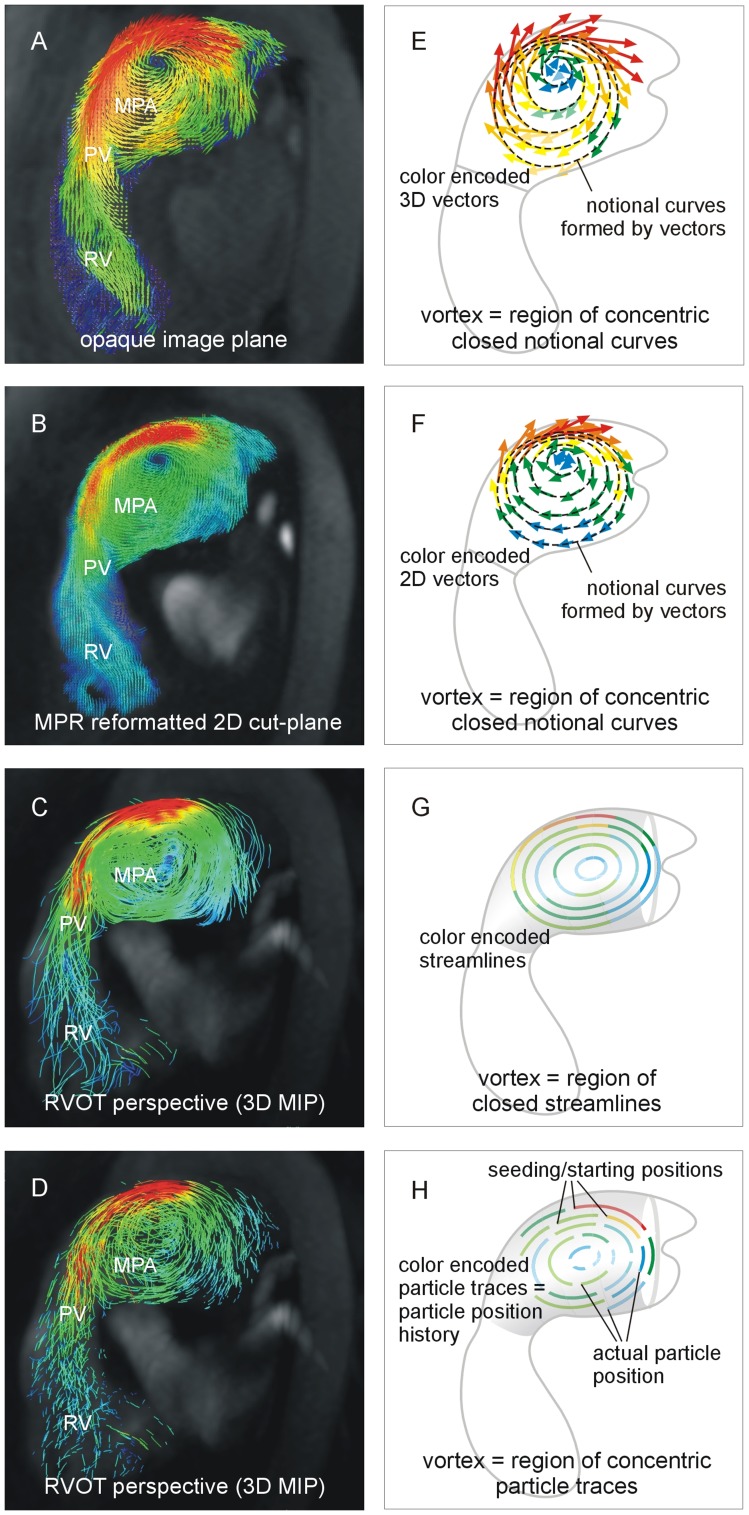
PH-associated vortical blood flow in the main pulmonary artery (MPA) in 3D vector (A), MPR vector (B), streamline (C) and particle trace (D) visualization together with schematic drawings of identification criteria of this flow pattern in 3D vector (E), MPR vector (F), streamline (G) and particle trace (H) visualization. PV: pulmonary valve, RV: right ventricle.

With 3D vector visualization, the magnitudes (with color encoding) and directions of the measured velocities are projected onto opaque anatomical images (Figure 1A). Through-plane components of velocities can be assessed by adjusting the opacity of anatomical images as well as the spatial rotation of image planes joined with 3D vectors. MPR vector visualization enables reconstruction of arbitrary cross-sectional planes in volume, showing interpolated in-plane velocity vectors projected onto multi-planar reformatted, anatomical images (Figure 1B). In our study, the magnitude and direction of in-plane velocities were displayed as the magnitude (enhanced with color coding) and direction of in-plane velocity vectors. For visualization of streamlines (Figure 1C) and particle traces (Figure 1D), starting points (“seeding” points) were chosen that were uniformly distributed in the segmented volume in each cardiac phase. Particle trace length was adapted to the seeding period to obtain continuous particle traces of adequate length. Magnitudes of velocities along streamlines and particle traces were further enhanced by color encoding. Projection into opaque 3D phase contrast angiographic reconstructions of PC-MRI data provided anatomical and perspective context.

Blood flow patterns were analyzed visually with respect to the presence and duration of PH-associated vortical motion of blood in the main pulmonary artery. A PH-associated vortex was defined as non-valvular rotational blood flow in the RVOT orientation. In 3D vector and MPR vector visualization, rotational blood flow was identified from the existence of notional, smooth, closed concentric tangent curves in the velocity vector field (Figure 1E and 1F). With streamline visualization, rotational blood flow was detected when closed streamlines were observed (Figure 1G), whereas with particle trace visualization, rotational blood flow was identified as a closed ring of particle traces with particle velocities not vanishing along the entire paths (Figure 1H).

If a PH-associated vortex was identified in a patient, its duration (t_vortex_), its time of onset (t_start_) and its time of termination (t_end_) were specified. t_vortex_ was defined as the duration of PH-associated vortical blood flow (divided by the cardiac interval and given as a percentage); t_start_ was defined as the time interval from pulmonary valve opening (derived from 3D vector visualization) to the onset of PH-associated vortical blood flow (divided by the cardiac interval and given as a percentage); and t_end_ was defined as the time interval from pulmonary valve opening to termination of PH-associated vortical blood flow (divided by the cardiac interval and given as a percentage).

### Statistical Analysis

Mean values are given together with standard deviations. Statistical analysis was performed using NCSS (Hintze, J. (2008) NCSS, LLC. Kaysville, UT, USA). For statistical tests a significance level of 0.05 was employed.

Cohen’s Kappa coefficient (κ) was calculated to specify interobserver agreement with respect to the detection of PH-associated vortices. Interobserver variability levels in the determination of t_vortex_, t_start_ and t_end_ were specified as within-subject standard deviations in variance component analysis together with the intraclass correlation coefficients r_IC_.

Further analyses were performed using t_vortex_, t_start_ and t_end_ values derived by the two readers in consensus. Agreement of 3D vector visualization with MPR vector, streamline and particle trace visualization with respect to the detection of PH-associated vortices was calculated as Kendall’s τ_B_ with corrections for ties. Symmetry was analyzed by using McNemar’s test. Comparison of t_vortex_, t_start_ and t_end_ measurements derived from 3D vector, MPR-vector, streamline, and particle trace visualization was performed by means of Pearson’s correlation coefficient r and Bland-Altman analysis.

The diagnostic performance of t_vortex_ in predicting PH from the various visualization techniques was investigated by means of receiver operating characteristic (ROC) curve analysis. Empirical areas under ROC curves (AUCs) describing the diagnostic performance of t_vortex_ as detected by MPR vector, streamline and particle trace visualization were compared to the empirical AUC describing the diagnostic performance of t_vortex_ detected by 3D vector visualization by z test. The relationship of t_vortex_ to mPAP was analyzed by means of correlation and linear regression analysis. Comparison of correlation coefficients between mPAP and t_vortex_ determined from 3D vector, MPR vector, streamline and particle trace visualization was performed by Williams-Hotelling test.

## Results

### Interobserver Variability of Vortex Detection

Interobserver variability levels in the measurement of t_vortex_ from 3D vector, MPR-vector, streamline and particle trace visualization were 2% (r_IC_ = 0.99), 4% (r_IC_ = 0.97), 5% (r_IC_ = 0.96) and 4% (r_IC_ = 0.97) of RR-interval, respectively. Interobserver agreement in the identification of patients with PH-associated vortices was high for 3D vector (κ = 0.88) and particle trace (κ = 0.87) visualization and medium for MPR vector (κ = 0.48) and streamline (κ = 0.59) visualization.

Both observers found PH-associated vortices in all patients with PH with all visualization techniques, so t_start_ and t_end_ of PH-associated vortical blood flow could be compared. Interobserver variability levels in the determination of t_vortex_, t_start_ and t_end_ in patients with PH are presented in Table 2.

**Table 2:** Interobserver variability in determination of t_vortex_, t_start_ and t_end_ from different flow visualizations in patients with PH (n = 23).

visualization	t_vortex_	t_start_	t_end_
**3D vector**	2% (0.99)	1% (0.99)	1% (1.00)
**MPR vector**	4% (0.96)	2% (0.95)	5% (0.89)
**streamline**	6% (0.91)	5% (0.75)	4% (0.91)
**particle trace**	6% (0.91)	4% (0.85)	6% (0.81)

Within-subject standard deviations are given together with intraclass correlation coefficients (in parentheses).

### Comparison of Flow Visualization Techniques

**Figure 2:**
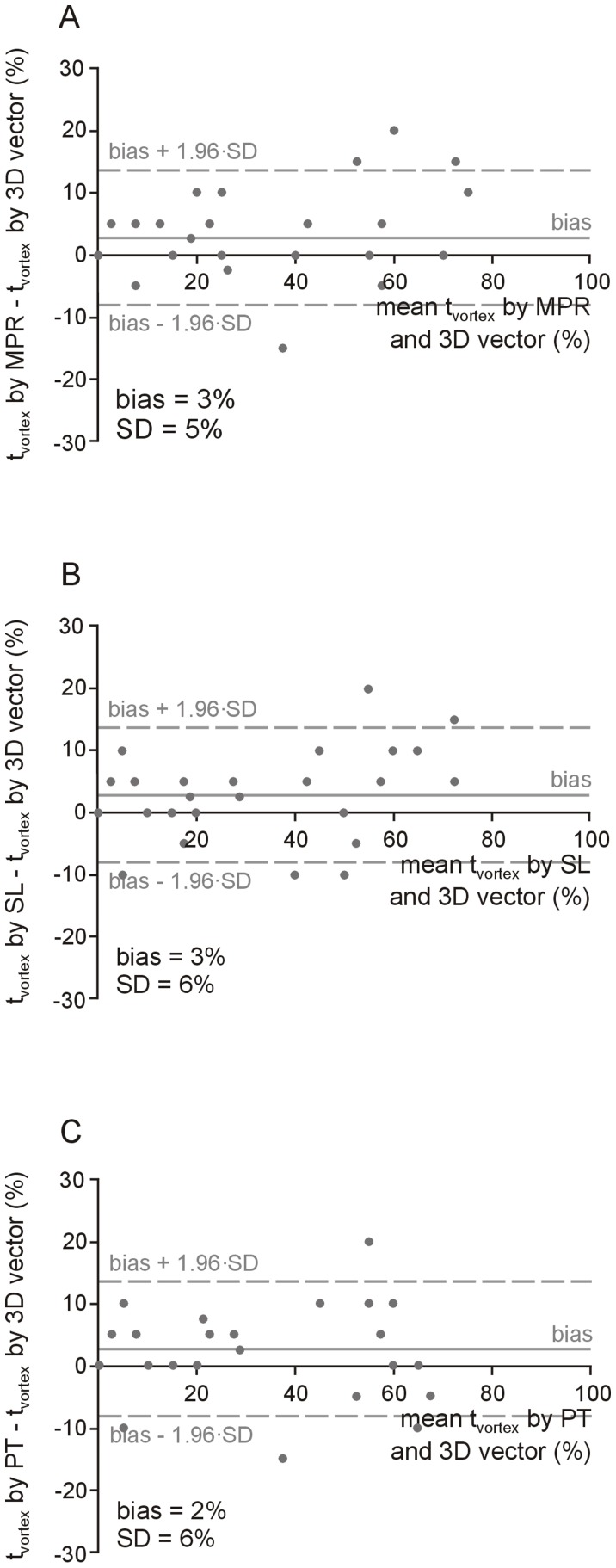
Bland-Altman plots of vortex duration (t_vortex_) determined from MPR vector (A), streamline (B) and particle trace (C) visualization compared to t_vortex_ determined by 3D vector visualization for all patients (n = 50). SD denotes standard deviation of measurement differences.

t_vortex_ determined by MPR vector, streamline and particle trace visualization correlated strongly with t_vortex_ derived from 3D vector representation (r = 0.98, 0.98 and 0.97, respectively). t_vortex_ determined from MPR vectors, streamlines and particle traces were, however, slightly larger than t_vortex_ determined from 3D vectors, with a standard deviation of measurement differences of 5–6% of the RR-interval (Figure 2).

Compared to the agreement on t_vortex_ observed between 3D vector visualization and the various other visualization techniques, agreement on the identification of patients with PH-associated vortical blood flow between 3D vector visualization and MPR vectors, streamlines and particle traces was weaker (τ_B_ = 0.71, 0.62 and 0.77, respectively). PH-associated vortices were identified in more subjects with analysis of MPR vector, streamline and particle trace visualization than with 3D vector visualization (p = 0.005, 0.011 and 0.102, respectively).

Mean values of t_vortex_, t_start_ and t_end_ in patients with PH are summarized in Table 3. As presented in Table 4, correlations between t_vortex_ measurements determined by different visualization techniques were lower in patients with PH than in all patients. Moreover, t_start_ and t_end_ of PH-associated vortical blood flow showed considerable dependence on visualization technique.

**Table 3:** Mean values and standard deviations of t_vortex_, t_start_ and t_end_ derived from different flow visualizations in patients with PH (n = 23).

visualization	t_vortex_	t_start_	t_end_
**3D vector**	40±18%	24±11%	65±16%
**MPR vector**	44±20%	23±10%	67±15%
**streamline**	44±21%	26±10%	71±15%
**particle trace**	43±18%	37±10%	80±12%

**Table 4:** Comparison of t_vortex_, t_start_ and t_end_ determined from MPR-vector, streamline and particle trace visualizations with the same parameters determined from 3D vector visualization in patients with PH (n = 23).

Comparison	t_vortex_	t_start_	t_end_
**MPR vector vs** **3D vector**	0.93 (4%/7%)	0.80 (−1%/7%)	0.76 (3%/11%)
**streamline vs** **3D vector**	0.94 (4%/7%)	0.65 (2%/8%)	0.82 (7%/9%)
**particle trace vs** **3D vector**	0.92 (3%/8%)	0.70 (12%/9%)	0.74 (16%/11%)

Given numbers are Pearson correlation coefficients as well as (in parentheses) bias and standard deviations of measurement differences as percentages of RR-intervals, resulting from Bland-Altman analysis. A negative bias means a larger mean value on 3D vector visualization.

### Non-invasive Diagnosis of PH based on Vortex Duration

**Figure 3:**
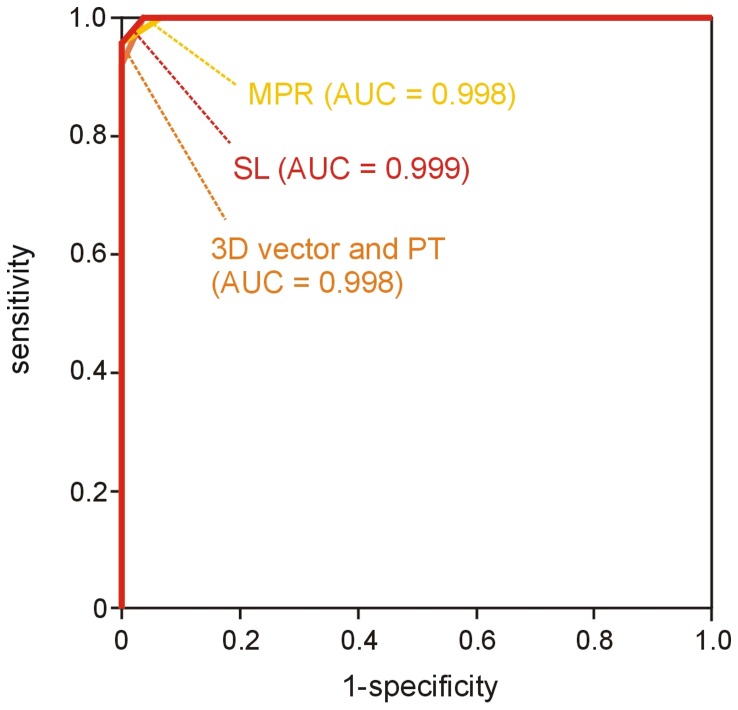
ROC curves for the diagnosis of manifest PH employing vortex duration (t_vortex_) determined from 3D vector, MPR vector, streamline and particle trace visualization.

Areas under ROC curves for non-invasive diagnosis of PH from t_vortex_ values from the various visualization techniques did not differ significantly. The AUCs were 0.998 (95% confidence interval [CI], 0.983–1.000) for 3D vector visualization, MPR vector visualization, and particle trace visualization and 0.999 (95% CI, 0.987–1.000) for streamline visualization (Figure 3). Optimal cut-off values of t_vortex_ for maximizing the sum of sensitivity and specificity are given in Table 5.

**Table 5:** Cut-off values of t_vortex_ determined from 3D vector, MPR-vector, streamline and particle trace visualizations for non-invasive diagnosis of PH.

visualization	t_vortex_ cut-off	sensitivity	specificity
**3D vector**	15%	1.00 (0.86 to 1.00)	0.96 (0.82 to 0.99)
**MPR-vector**	20%	0.96 (0.79 to 0.99)	1.00 (0.88 to 1.00)
**streamline**	15%	1.00 (0.86 to 1.00)	0.96 (0.82 to 0.99)
**particle trace**	15%	1.00 (0.86 to 1.00)	0.96 (0.82 to 0.99)

**Figure 4:**
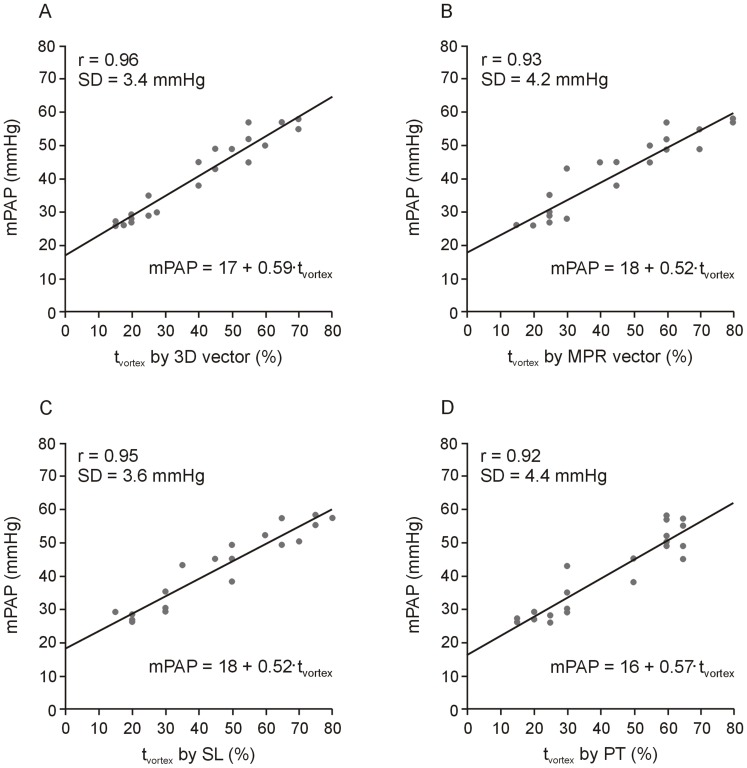
Scatter plot and linear regression lines of mPAP and vortex duration (t_vortex_) determined from 3D vector (A), MPR vector (B), streamline (C) and particle trace (D) visualization for patients with PH (n = 23). Regression equations are to be understood in mmHg. SD denotes standard deviation from regression line.

% confidence intervals (in parentheses). Cut-off values were chosen to maximize the sum of sensitivity and specificity. Sensitivity and specificity are given with 95

t_vortex_ correlated strongly with mPAP for patients with PH (r = 0.96 in case of 3D vector, r = 0.93 in case of MPR vector, r = 0.95 in case of streamlines, and r = 0.92 in case of particle trace visualization) and the correlations did not differ significantly. Linear regression lines between mPAP and t_vortex_ values derived from 3D vector, MPR vector streamline and particle trace visualization are shown in Figure 4.

## Discussion

The present study showed that 3D vector, MPR vector, streamline and particle trace visualization techniques for analyzing PH-associated vortical blood flow in the main pulmonary artery on 3D PC-MRI all allow highly accurate diagnosis of PH and estimation of elevated mPAP. However, interobserver variability in vortex identification was smallest with 3D vector visualization.

A previous study employing 3D vector field representation of PC-MRI data established a linear relationship between PH-associated vortical blood flow in the main pulmonary artery and elevated mPAP [3]. In agreement with the findings of the earlier study, the present study found low inter-observer variability in vortex detection, high sensitivity and specificity of PH diagnosis based on t_vortex,_ and strong linear correlation between t_vortex_ and elevated mPAP with 3D vector visualization. Additionally, ROC analysis allowed the specification of an optimal cut-off value of t_vortex_ = 15% for non-invasive diagnosis of PH (mPAP≥25 mmHg).

t_vortex_ values determined from MPR vector, streamline and particle trace visualization correlated strongly with those derived from 3D vector representation, and correlations between elevated mPAP and t_vortex_ values derived from 3D vector, MPR vector, streamline and particle trace visualization did not differ significantly. Cut-off values, as well as linear regression equations and standard deviations for prediction of elevated mPAP were rather similar in all visualization techniques (Figure 4), indicating that all of the techniques tested are appropriate for non-invasive diagnosis of PH.

Numbers of patients with PH-associated vortices, as well as t_vortex_ values, were larger with the use of MPR vector, streamline and particle trace visualization techniques than when 3D vector visualization was used. Furthermore, interobserver variability of t_vortex_ was greater with the former three visualization techniques than with 3D vector analysis. These differences can be explained by the differing abilities of the particular visualization techniques to display and separate complex dynamic blood flow patterns such as valvular-vortical, bifurcation-related, helical, and PH-associated vortical blood flow [18]–[21].

**Figure 5:**
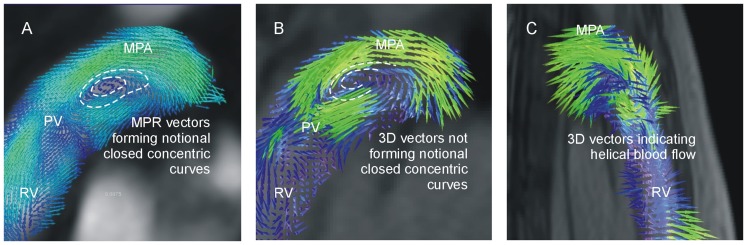
Helical blood flow in the main pulmonary artery mimicking PH-associated vortical blood flow in MPR vector representation (A). The same perspective in 3D vector visualization is not interpreted as showing a PH-associated vortex (B). Rotation of perspective (C) indicates the helical character of the blood flow. RV: right ventricle, PV: pulmonary valve, MPA: main pulmonary artery.

Interobserver agreement on the presence of PH-associated vortical blood flow was lowest with MPR vector visualization. MPR vectors can be reformatted on arbitrary image orientations in volume, which is helpful to optimize the RVOT view but causes higher variability in visualized patterns because of the observer-dependent angulation of reconstruction. Additionally - and even more importantly - MRP vector visualization, representing 2D in-plane velocities projected onto anatomical images, neglects through-plane components of 3D velocities, impeding differentiation of helical from vortical blood flow. Helical blood flow in the main pulmonary artery, frequently observed in healthy volunteers as well as patients, has been related to anatomy, curvature and contraction characteristics of the right ventricle and outflow tract [20], [22], [23]. As shown in Figure 5, these flow patterns might lead to misinterpretation of helical as PH-associated vortical blood flow in MPR vector visualization. Moreover, beginning in late systole, helical motion of blood does not severely affect definition of t_start_ but hinders identification of t_end_ and therefore limits the validity of t_vortex_ from MPR vector visualization (Table 2 and 4).

**Figure 6:**
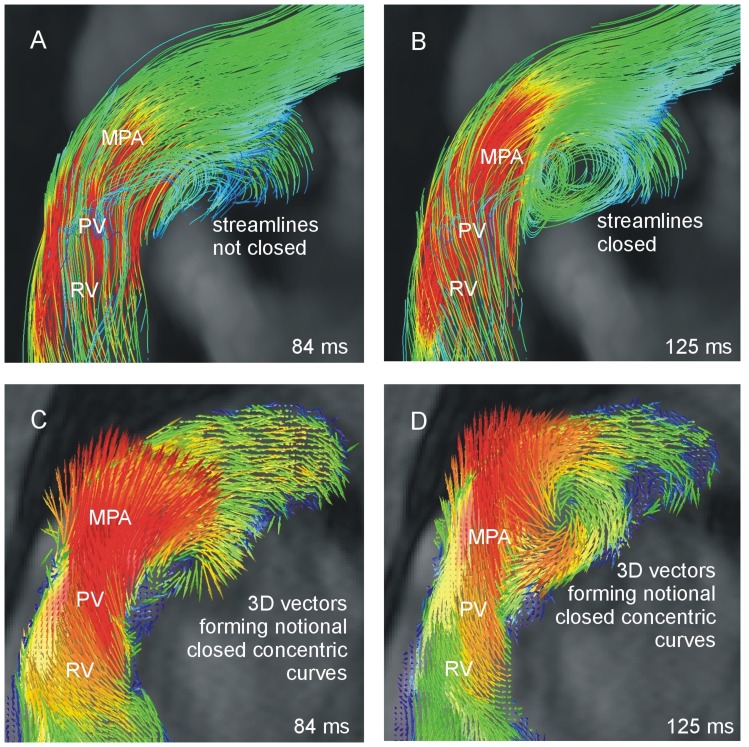
Blood flow patterns in the main pulmonary artery of a patient with PH in streamline (A, B) and 3D vector visualization (C, D) in two consecutive systolic cardiac phases. Onset of vertical blood flow t_start_ is defined later in streamline (B) than in 3D vector visualization (C). RV: right ventricle, PV: pulmonary valve, MPA: main pulmonary artery.

In the current study interobserver variability in streamline visualization was moderate; furthermore, in determining which patients had PH-associated vortices, streamline visualization agreed with 3D vector visualization less often than any other visualization technique investigated. Both results can be explained by the fact that notional curves of velocity vectors, synonymously notional streamlines, are subject to some kind of visual filtering: A single closed streamline, or a few concentric calculated streamlines, which might already be assigned as PH-associated vortical blood flow, will often not be noticed in vector representation. This also explains the larger t_vortex_ values and later t_ends_ values found in streamline compared to vector visualization. The fact that t_start_ was generally detected later in streamline than vector visualization relates to the typical behavior of streamlines: that is, uniformly distributed seeding points do not result in evenly spaced streamlines but in clusters in regions with high velocities, and appear low-density in low-velocity regions [5], [24]. As the onset of PH-associated vortical blood flow typically starts in systole (Table 3) during high-velocity blood flow in the main pulmonary artery, it may easily happen that small regions of closed streamlines are occluded or underrepresented and therefore missed. An example of t_start_ being detected later in streamline than in 3D vector visualization is shown in Figure 6.

As particle traces observed in a specific cardiac phase are trajectories of particles seeded in previous times, they do not necessarily coincide with streamlines in time-varying velocity fields [12], [25], [26]. The strong correlation between t_vortex_ in particle trace and 3D vector visualization first of all confirms that RVOT-oriented, ring-shaped blood circulation in the main pulmonary artery (the definition of a PH-associated vortex in particle trace visualization) corresponds to a PH-associated vortex observed in vector and streamline visualization. The striking difference between PH-associated vortices detected in particle trace and those detected in 3D vector visualization was that onset and termination of vortical blood flow was identified substantially later in particle trace visualization. This result can be explained by the fact that 1) particles were defined to have zero-length in the first time-frame, 2) seeded particles need some time to cover distances long enough to be identified as a ring-shaped particle trace (in particular when velocity is low), and 3) long-living vortices observed at very high mPAP might last until the next cardiac cycle, which was not considered in the current particle trace implementation. The high t_vortex_ values shown in Figure 4D might therefore be slightly underestimated.

In the present study, there were limitations with respect to data acquisition and visualization that will need to be addressed. Underestimation of high mPAP values from particle trace analysis due to underrating of t_vortex_ could be prevented by cyclic particle seeding, which was not performed. In general the results for streamlines and particle traces are limited to standard 3D integral curve visualizations provided by the employed 4D Flow prototype software, and additional techniques such as opacity, illumination or topology passed seeding strategies might further improve non-invasive mPAP estimation [5]–[7], [24]. In order to cover the main pulmonary artery in reasonable imaging time, PC-MRI data were acquired with 6-mm slice thickness and measured time resolution of 89 ms. Although spatially interpolated, calculation of streamlines and particle traces might be limited due to the anisotropic 3D velocity field acquired. The moderate time resolution might have furthermore influenced the precision of calculated particle traces. Finally, performance of 3D vector visualization was privileged by the coincidence of the chosen imaging and the rotational plane of PH-associated vortices.

In conclusion, 3D vector, MPR vector, streamline and particle trace visualization of time-resolved 3D PC-MRI data of the main pulmonary artery can be employed for accurate vortex-based diagnosis of PH and estimation of elevated mPAP. As all visualizations have advantages and drawbacks, their combined usage might contribute to a refined understanding and an establishment of automatic extraction of topological features of blood flow patterns in the main pulmonary artery.
